# Infertility as a Proxy of Men’s Health: Still a Long Way to Go

**DOI:** 10.5152/tud.2021.20561

**Published:** 2023-03-01

**Authors:** Edoardo Pozzi, Luca Boeri, Paolo Capogrosso, Luigi Candela1, Walter Cazzaniga, Federico Belladelli, Antonio Costa, Daniele Cignoli, Costantino Abbate, Francesco Montorsi, Andrea Salonia

**Affiliations:** 1 Division of Experimental Oncology/Unit of Urology; URI; IRCCS Ospedale San Raffaele, Milano, Italy; 2 Department of Urology, Foundation IRCCS Ca’ Granda-Ospedale Maggiore Policlinico, University of Milano, Milano, Italy; 3 Department of Urology and Andrology;, Ospedale di Circolo and Macchi Foundation, Varese Italy; 4 University Vita-Salute San Raffaele, Milano, Italy

**Keywords:** Body mass index, cancer, diabetes, health, male infertility, mortality

## Abstract

Male infertility (MI) has been widely associated with the development of certain comorbidities and to a lower overall general health status. Higher risks of developing oncological, autoimmune, and chronic disorders among infertile individuals have led researchers to further investigate this issue. Recent clinical studies have been focusing more onto the concept of general health status and mortality. Overall, it has been postulated and subsequently demonstrated that the coexistence of specific diseases and semen alterations may lead to a decreased lifespan. As in Western countries, fatherhood is increasingly delayed in time, and aging might play an important role as a confounding factor for the after-mentioned statements. Although this holds true, even after adjusting for age, it emerges a worrisome picture regarding MI, lower general health status, and increased mortality. The aim of this nonsystematic narrative review is to provide an overview of the most relevant and recent findings on the topic.

**Main Points**

- Data suggest that certain population of men suffering from infertility might have their lifespan reduced, compared with fertile controls.
- Findings almost unanimously confirm how infertile patients sometimes display precarious health, as a consequence of the collection of coexistent diseases.
- It emerges how assessing patient’s health status is of primary importance in the setting of male infertility.
- Additional studies in larger population-based samples are needed to confirm these findings and to further characterize the potential link between male infertility and decreased lifespan.

## Introduction

The association between a lower general health status and male infertility (MI) has been widely studied and established.^1^ As a matter of fact, certain comorbidities have been found to bidirectionally correlate with MI.^2^,^3^ The cardinal example of this is the 20-fold increased risk of developing testicular cancer among infertile men with respect to a same-age- and racematched group of fertile men from the general population. In addition, other oncological malignancies have been found to be intertwined with male MI as colorectal cancer, melanoma, and prostate cancer (PCa).^2^,^4^

Likewise, systemic and urogenital infections, autoimmune disorders, endocrinopathies, and chronic kidney/liver diseases have all been ascribed to negatively affect semen parameters.^5^-^10^

Moreover, over time, studies have focused more about the comprehensive concept of MI and general health status. In fact, it has been demonstrated how infertility is linked with lower overall well-being and increased mortality with respect to fertile individuals.^1^-^4^,^11^

Recently, it has been surveyed and discovered that young men tend to delay fatherhood with respect to the past. In this context, the age range of 34–40 years is becoming more and more likely to be chosen by young adults to father a child.^12^ In line with this, sperm alterations are, in all likelihood, to occur during that period of a man’s life (34–40 years), thus having possible detrimental effects on conceiving a child.^12^,^13^ As certain diseases also affect semen parameters,^6^,^7^,^13^-^15^ these findings depict a worrisome picture showing a vicious cycle that dramatically affect the chances of fatherhood. Based on these premises, it emerges how assessing patient’s health status is of primary importance in the MI setting. Moreover, data demonstrate that impaired semen parameters can predict mortality, suggesting that semen analysis may represent a potential and possible biomarker of overall health and fitness. This narrative review gathers findings on general health status and MI, summarizing past and latest findings on this evolving and relevant topic.

## Methods

The PubMed database was used for research of English-language articles published up to November 2020. This nonsystematic narrative literature review primarily focuses on studies published in the context of MI as a proxy of general health status. Older articles closely related to this topic were also included.

### Male infertility and oncological malignancies

Consistent evidence over the past few decades has shown a higher prevalence of malignant diseases among patients with MI as compared with their fertile counterparts.

### Testis cancer

The association between testicular germ cell tumors (TGCTs) and MI is one of the most comprehensively investigated association. Small case-control studies initially reported controversial findings regarding the relationship between MI and TGCTs,^16^,^17^ and a subsequent meta-analysis of case-control studies found a 3-fold higher risk for testis cancer among patients with infertility.^18^ However, the overall level of evidence of the analyzed findings was low. Larger studies using national registry data confirmed these findings. A European case-control study analyzed data of 4,592 men with testis cancer compared to 12,254 controls and showed a lower risk of cancer in men who had fathered children.^19^ Similarly, Baker et al. analyzed US population comparing men with testis cancer and age-matched controls.^20^ The authors showed that men with testis cancer were less likely to have fathered children compared with controls, and they were more likely diagnosed with infertility (Odds Ratio=9.47; 95%CI: 1.19–75.2). Major limitation of the previous studies was that fathering was considered a surrogate for fertility, which is not in line with current World Health Organization (WHO) definition.

Even more robust pieces of evidence have been provided from population-based cohort studies.^21^-^24^ Raman et al.^21^ retrospectively assessed the incidence of TGCTs among 3,800 infertile patients by linking their data to that from regional cancer registries and to the average rate of testis cancer from the Surveillance, Epidemiology and End Results (SEER) database. The authors found only 10 men with subsequent cancer diagnosis, and the standardized incidence ratio (SIR) was 22.9 (95% CI: 22.4–23.5) compared with the SEER population. In a larger study examining 32,442 Danish men undergoing semen evaluation, the SIR of TGCT was 1.6 (95% CI: 1.3–1.9) for infertile men compared with the general population.^22^ Of note, the authors reported that semen alterations (e.g., poor motility, altered morphology, and low semen concentration) were significantly associated with a diagnosis of TGCTs. Walsh et al.^23^ conducted a multicenter study, including 51,461 couples recruited from 15 centers in California, to assess the incidence of testicular cancer among male partners and compared results with data from the SEER database. They showed that infertile men had a 3-fold higher risk of testis cancer compared with fertile controls. These findings were further confirmed in a recent retrospective study, showing that men with semen alterations had an increased risk of testis cancer with a hazard ratio (HR) of 3.3 compared with controls; the association was even stronger for patients with oligozoospermia.^24^ These findings have been recently summarized in a systematic review and meta-analysis of population-based retrospective cohort studies that showed a 2-fold increase in relative risk (RR) of development of testis cancer among fertility-impaired males (RR 2.03; 95% CI: 1.66–2.48).^25^

### Prostate cancer

Several studies have investigated the association between MI and PCa. In 2010, Walsh et al.^23^ were among the first to report data on the incidence of PCa among infertile men. By using data from the multicenter California infertility dataset and the California Cancer Registry, the authors found that the incidence of PCa after the diagnosis of MI was comparable with the general population. However, infertile men showed a significantly higher incidence of high-grade disease.^23^ Similarly, Eisenberg et al.^26^ analyzed 76,083 infertile men and reported a higher risk of PCa (HR=1.78; 95% CI: 1.41–2.25) compared with control populations. Recently, Al-Jebari et al.^27^ have compared the risk and severity of PCa between men achieving fatherhood by assisted reproduction and men conceiving naturally. The authors found that men who became fathers through assisted reproduction had a statistically significantly increased risk of PCa as compared with men who conceived naturally (HR=1.64 and 95% CI: 1.25–2.15 for intracytoplasmic sperm injection; HR=1.33 and 95% CI: 1.06–1.66 for *in vitro* fertilization) along with an increased risk of early onset disease. These findings have been recently summarized in a meta-analysis of population-based retrospective cohort studies that showed a pooled RR of 1.68 (95% CI: 1.17–2.4) for PCa for infertile men compared with fertile controls.^25^

Of note, other authors did not corroborate these data. Using a nested case control design, Ruhayel et al.^28^ showed that men with PCa had a lower rate of MI as compared with fertile controls (OR=0.45; 95% CI: 0.25–0.8). The study from Hanson et al.^24^ on subfertile American men from the Subfertility Health and Assisted Reproduction study and the Utah Cancer Registry did not identify a difference between subfertile men and controls with regard to PCa risk. In this case, however, the majority of men in the sample had not reached the age normally associated with PCa. Of note, a meta-analysis of 18 earlier epidemiologic studies failed to confirm the observed inverse association between fatherhood and PCa, likely due in part to the heterogeneity of the infertility definition.^29^ More recently, Boeri et al.^30^ have investigated prostate-specific antigen (PSA) values in 956 men presenting for primary couple’s infertility as compared with a cohort of 102 fertile individuals, according to the recommendation of the European Association of Urology guidelines that a first PSA assessment should be done at 40–45 years of age. The authors found that infertile men have higher PSA values than fertile individuals, and that a greater proportion of infertile men (approximately 30%) younger than 40 years had total PSA>1 ng/mL at the first assessment. Hence, considering the known association between MI and a greater risk of PCa, the authors speculated that infertile men may deserve further attention and comprise an easily accessible category of patients who may eventually benefit from early PCa screening with PSA testing.^30^

### Other malignancies

Male factor infertility has also been associated with nonurological malignancies. In a cohort study, including infertile, fertile, and patients who underwent vasectomy, Eisenberg et al.^26^ showed that patients with MI had a 49% higher risk for being subsequently diagnosed with any cancer (HR=1.49; 95% CI: 1.37–1.63) compared with fertile men, thus considering melanoma, bladder and thyroid cancer, as well as hematological malignancies. Of note, a lower but significantly higher risk of cancer was also detected for the post-vasectomy group compared with controls. Finally, in a study of 2,238 infertile men linked to the Texas Cancer Registry, the authors assessed the association between azoospermia and the risk of cancer (any type).^4^ Men with azoospermia had a 2.2-fold higher risk of cancer compared with nonazoospermic men.

The possible etiological link between MI and the subsequent risk of malignancy is far from being elucidated. Previous evidence suggested that men with reproductive health disorders may lack regulatory genes that predispose them not only to abnormal spermatogenesis but also to abnormal control mechanisms for cell division and an increased probability of cancer.^2^,^4^,^19^,^23^ Similarly, variations in the number of cytosine-adenine-guanine (CAG) repeats in genes encoding for the androgen receptor, mutations in DNA repair genes, and epigenetic and environmental modulators have also been suggested to link MI and PCa.^31^-^33^

### Male infertility, metabolic, autoimmune, and chronic disorders

Specific conditions included in the definition of metabolic syndrome (MetS) (co-existence of three or more of the following: fasting plasma glucose ≥110 mg/dL, serum triglycerides ≥150 mg/dL, serum highdensity lipoprotein (HDL) cholesterol <40 mg/dL, BP ≥130/85 mmHg or on BP medication, or waist girth >102 cm) have been found to be intertwined with MI.^7^,^34^-^37^ In this context, data from three large-scale epidemiological studies suggested that overweight and/or obese men have altered semen parameters and difficulties in fathering a child.^7^,^38^ Additionally, other studies have confirmed the inverse correlation between body mass index (BMI) and total sperm count.^38^ The pathophysiological mechanism behind these alterations relies on the fact that obesity, insulin resistance, and diabetes mellitus (DM) negatively influence androgen levels via the downregulation of serum levels of sex hormone binging globulin (SHBG).^39^ In this context, the European Male Ageing Study (EMAS) found that 73% of men with reduced testosterone (T) were overweight or obese. Strengthening this, another study of the EMAS and a meta-analysis demonstrated that weight gain suppresses, and weight loss increases, serum T levels.^40^,^41^ Of further note, overweight men have increased estradiol (E2) levels, thus resulting in reduced T/E2 ratio. Low serum T/E2 ratios are often seen among infertile men and have been found to adversely affect spermatogenesis.^42^-^44^ As a matter of fact, obesity, aging, and the onset of chronic diseases (e.g., DM) should all be considered when T levels are suppressed as these conditions are all entwined with male factor infertility.^35^,^41^ Confirming this, a recent study has shown that oligoteratoasthenospermic patients with MetS treated with metformin for 6 consecutive months reported improvements in hormone, metabolic, and, above all, semen characteristics.^45^ Subsequently, Wang et al.^46^ used an IBM MarketScan database investigating 13,000 infertile men; the group found a significant association between the presence of altered semen parameters and the development of type-2 DM, alcohol abuse, and drug abuse (HR=1.30 and 95% CI: 1.10–1.53; HR=1.48 and 95% CI: 1.07–2.05; and HR=1.67 and 95% CI: 1.06–2.63, respectively) compared with men who had only undergone fertility testing. Likewise, a very recent study from Ferlin et al. has found that that poor semen quality itself emerged as a biomarker of poor general health, regardless of the presence of hypogonadism. Men with low sperm count had higher BMI, waist circumference, systolic pressure, low-density lipoprotein cholesterol, triglycerides, insulin resistance, and lower HDL cholesterol than men with a normal sperm count.^47^ Furthermore, the authors found that men with lower sperm counts were also at a higher risk of hypogonadism (OR=12.2; 95% CI: 10.2–14.6).^47^ In line with this, Salonia et al.^2^ were the first to assess whether men with male factor infertility were less healthy than fertile men, as objectively scored with an internationally validated and reliable hospital-based comorbidity index (Charlson Comorbidity Index [CCI]), regardless of the reasons for infertility. The group evaluated 344 consecutive European Caucasian men with male factor infertility and demonstrated a higher prevalence of comorbidities compared with fertile controls (CCI: 0.33 [0.8] versus 0.14 [0.5], p<0.001; 95% CI: 0.08–0.29). Although 88.4% of the fertile controls had a CCI=0, only 77.3% of the infertile men had CCI=0 (p<0.001). Moreover, at multivariable linear regression analysis, age, BMI, and fertility status were all found to independently predict CCI scores with all p<0.001.^2^ Likewise, Ventimiglia et al.^3^ analyzed complete demographic, clinical, and laboratory data from 2,100 consecutive infertile men with health-significant comorbidities scored via the CCI and semen analysis values based on 2010 WHO reference criteria. They offered novel and updated evidence that patients with a decreased general health status have lower sperm concentration, lower T levels, and higher follicle stimulating hormone (FSH) values, confirming that poor health status appears to be associated with a malfunctioning male reproductive system. Eisenberg et al.^48^ have recently observed that by stratifying their large cohort of infertile men according to the CCI, men with diseases of the endocrine, circulatory, or genitourinary systems or skin diseases all showed significantly higher rates with semen abnormalities. Finally, autoimmune disorders-such as systemic lupus erythematosus, psoriasis, thyroiditis, and Grave’s disease-were all found to be associated after the analysis of 33,077 infertile men taken from the IBM Market Scan database (2001–2008).

### Male infertility and increased mortality

Finally, it has also been postulated and subsequently demonstrated that infertile men have increased mortality with respect to the general population. In this context, a large Swedish cohort of men with MI was analyzed, and the authors found that cancer mortality was higher in men with a diagnosis of infertility and in those with an infertility-related diagnosis. However, cancer mortality was only higher in those individuals with a diagnosis of cancer before MI diagnosis.^49^ Of note, in this study, the most common cancer types registered among infertile men were brain tumors, hematological cancers, and tumors of bone, cartilage, mesothelium, and soft tissue.^49^ Likewise, Eisenberg et al.^50^ investigated 11,935 men with MI from 1989 to 2011; first, as compared with the general population, men evaluated for infertility had a lower risk of death with 69 deaths observed compared with 176.7 expected (standardized mortality rate=0.39; 95% CI: 0.30–0.49). However, when stratified by semen parameters, men with impaired semen parameters had significantly higher mortality rates as compared with men with normal parameters. Low semen volume, sperm concentration, sperm motility, total sperm count, and total motile sperm count were all associated with a higher risk of death. In contrast, abnormal sperm morphology was not associated with mortality. Finally, after adjusting for current health status, men with two or more abnormal semen parameters still had a 2.3-fold higher risk of death as compared with men with normal semen (95% CI: 1.12–4.65).^50^ In conclusion, in a recent study, Glazer et al.^51^ have investigated the risk of death among men with oligospermia, unspecified male factor infertility, and azoospermia; using national health registers, the authors found an increased risk of death among azoospermic men, while no increased risk was registered among men with other types of infertility. As a consequence, within azoospermic men, further insights into causal pathways are needed to identify options for monitoring and prevention.

## Discussion

Our review of the published literature shows that MI is unanimously linked with a lower general health status. On the one hand, the literature shows that obesity, autoimmune diseases, specific malignancies, and metabolic disorders (e.g., DM) are more common among men with altered semen parameters.^25^,^51^ On the other hand, these conditions negatively affect sperm characteristics making it sometimes difficult to distinguish which condition came first. In this context, some explanations have been proposed. Indeed, Ventimiglia et al. hypothesized two different mechanisms to explain the coexistence of infertility and comorbidities: (i) the existence of a common mechanism promoting both infertility and a particular subset of associated pathological conditions, and (ii) comorbidities that directly interfere with male reproductive function.^3^ The first hypothesis relies on the assumption that men with reproductive disorders lack specific genes, which are involved not only in ensuring correct spermatogenesis but also in guaranteeing impeccable cell division. If these are lacking or malfunctioning, spermatogenesis is, therefore, impaired, leading to the development of certain malignancies owing to the fact that cell division becomes increasingly imprecise. In this regard, DNA repair genes have been identified to regulate gamete formation.^52^ As such, polymorphisms in the MLH1 gene are frequently found in patients suffering from Lynch syndrome and have been linked to azoospermia^53^ In addition, the same polymorphism has been linked to an increased sperm DNA fragmentation index. Finally, preclinical data showed that a mice model lacking the ERCC1 gene (an important DNA repair gene) developed both azoospermia and cancer.^54^ Strengthening this hypothesis is the well-known association between cryptorchidism, testicular cancer, and altered semen parameters with data showing a strong association between delayed orchiopexy and an increased rate of cancer/infertility, thus clearly suggesting the key role of “*in situ* environmental factors.”^52^

The second hypothesis instead takes into consideration that some comorbidities have detrimental effects on male fertility. Although hormonal homeostatic changes (e.g., higher rates of hypogonadism) brought on by MetS (and obesity per se) have been widely reported and accepted,^55^-^57^ the effects on semen parameters are still inconclusive.^56^,^57^ In this context, recent findings from Boeri et al.^39^ have revealed a remarkably wide distribution of SHBG concentrations across age and BMI in primary infertile men. Of note, the authors found that the association between increasing BMI values and lowered SHBG concentrations emerged to be greater than the association of aging with increased SHBG.^39^ Likewise, findings on men suffering from diabetes have documented to alter semen parameters and spermatogenesis markers even though still not univocal.^58^ Even if data are somewhat inconsistent, the idea is that some comorbidities act together to dismay overall reproductive health.^50^,^59^ Finally, chronic liver diseases and autoimmune diseases have been found to alter semen quality and, therefore, should be taken into consideration for the overall clinical framework of men with MI. In conclusion, the interconnection between overall health and MI inevitably leads to consider specific diagnostic workups and adoption of tailored prevention strategies for men suffering from MI. The aim of the after mentioned strategies would be to prevent and promptly address specific comorbidities and to guarantee better fertility too.

## Conclusion

Overall, these data clearly show that MI is closely linked with the development of certain comorbidities. Compelling evidence has accumulated over the years with specific focus on overall general health status and increased mortality. Data suggest that certain population of men suffering from infertility might have their lifespan reduced, with respect to fertile controls. Although some studies report contrasting results, we cannot derive general conclusions regarding the increased mortality among patients with MI. These findings almost unanimously confirm how infertile patients sometimes display precarious health, as a consequence of the collection of coexistent diseases. Moreover, even after adjusting for age (which acts as a possible confounding factor), certain men with specific semen alterations (e.g., azoospermia) seem to have an increased mortality with respect to other groups of subfertile and fertile controls. Owing to these premises, it emerges how assessing patient’s health status is of primary importance in the setting of MI. Additional studies in larger population-based samples are needed to confirm these findings and to further characterize the potential link between MI and decreased lifespan.
